# DUDE - a universal prevention program for non-suicidal self-injurious behavior in adolescence based on effective emotion regulation: study protocol of a cluster-randomized controlled trial

**DOI:** 10.1186/s13063-021-05973-4

**Published:** 2022-01-31

**Authors:** Arne Buerger, Theresa Emser, Alexandra Seidel, Christin Scheiner, Cornelia von Schoenfeld, Viktoria Ruecker, Peter U. Heuschmann, Marcel Romanos

**Affiliations:** 1 Department of Child and Adolescent Psychiatry, Psychosomatics and Psychotherapy, Center of Mental Health, University Hospital of Wuerzburg, Margarete-Hoeppel-Platz 1, 97080 Wuerzburg, Germany; 2 German Centre of Prevention Research in Mental Health, University of Wuerzburg, Margarete-Hoeppel-Platz 1, 97080 Wuerzburg, Germany; 3 Institute of Clinical Epidemiology and Biometry, University of Wuerzburg, Josef-Schneider-Strasse, 97080 Wuerzburg, Germany

**Keywords:** Universal prevention, NSSI, Self-injury, Emotion regulation, RCT, School-based prevention, adolescence

## Abstract

**Background:**

Non-suicidal self-injury (NSSI) has become a substantial public health problem. NSSI is a high-risk marker for the development and persistence of mental health problems, shows high rates of morbidity and mortality, and causes substantial health care costs. Thus, there is an urgent need for action to develop universal prevention programs for NSSI before adolescents begin to show this dangerous behavior. Currently, however, universal prevention programs are lacking.

**Methods:**

The main objective of the present study is to evaluate a newly developed universal prevention program (“DUDE – Du und deine Emotionen / You and your emotions”), based on a skills-based approach in schools, in 3200 young adolescents (age 11–14 years). The effectiveness of DUDE will be investigated in a cluster-randomized controlled trial (RCT) in schools (*N* = 16). All groups will receive a minimal intervention called “Stress-free through the school day” as a mental health literacy program to prevent burnout in school. The treatment group (*N* = 1600; 8 schools) will additionally undergo the universal prevention program DUDE and will be divided into treatment group 1 (DUDE conducted by trained clinical psychologists; *N* = 800; 4 schools) and treatment group 2 (DUDE conducted by trained teachers; *N* = 800; 4 schools). The active control group (*N* = 1600; 8 schools) will only receive the mental health literacy prevention. Besides baseline assessment (T0), measurements will occur at the end of the treatment (T1) and at 6- (T2) and 12-month (T3) follow-up evaluations. The main outcome is the occurrence of NSSI within the last 6 months assessed by a short version of the Deliberate Self-Harm Inventory (DSHI-9) at the 1-year follow-up (primary endpoint; T3). Secondary outcomes are emotion regulation, suicidality, health-related quality of life, self-esteem, and comorbid psychopathology and willingness to change.

**Discussion:**

DUDE is tailored to diminish the incidence of NSSI and to prevent its possible long-term consequences (e.g., suicidality) in adolescents. It is easy to access in the school environment. Furthermore, DUDE is a comprehensive approach to improve mental health via improved emotion regulation.

**Trial registration:**

German Clinical Trials Register (DRKS) DRKS00018945. Registered on 01 April 2020, https://www.drks.de/drks_web/navigate.do?navigationId=trial.HTML&TRIAL_ID=DRKS00018945

## Background

### Non-suicidal self-injury

Non-suicidal self-injury (NSSI) has gained increasing importance in recent years, from both a clinical and socio-political perspective. According to the World Health Organization (WHO), self-injurious behavior represents the fifth most frequent health risk in adolescence [1], and it affects approximately 17–18% of adolescents worldwide [2]. NSSI is characterized by the intentional and self-inflicted destruction of body tissue without suicidal intent [3]. Above all, it is a high-risk marker for the development and persistence of mental health problems in adolescents, and has been highlighted by researchers all over the world. NSSI is a predictor of suicidal behavior [4, 5]; it is strongly associated with comorbid psychopathology (e.g., depression, anxiety disorders, posttraumatic stress disorder, borderline personality disorder) [6, 7] and other high-risk behaviors (e.g., substance abuse, suicidal thoughts) [8, 9]. Furthermore, NSSI causes substantial costs for the health system [10]. Given that NSSI is a common and highly recurrent behavior that peaks in adolescence around the age of 16 years [11, 12], there is an urgent need for action, especially in the area of universal prevention, prior to the age of 11 to 14 years when adolescents begin to show these dangerous behaviors.

### Universal prevention programs targeting NSSI

So far, universal prevention programs are lacking. Targeted prevention programs (*n* = 21) currently outnumber universal approaches (*n* = 2) and focus on adolescents with a history or current episodes of NSSI and mostly comorbid mental health problems [13].

Targeted approaches have been found to significantly reduce NSSI and improve mental health [14, 15]. However, access to these treatments is limited due to a lack of resources (e.g., effective interventions for adolescents addressing NSSI are not available across all regions) and an insufficient number of specially trained clinicians [16]. To the best of our knowledge, only two school-based universal prevention programs for adolescents are available to date [17, 18]. The signs of self-injury (SOSI) program by Muehlenkamp and colleagues [18] attempts to increase knowledge, improve help-seeking attitudes and behaviors, and decrease NSSI through the use of psychoeducational elements within one module for students and one module for faculty/staff. An uncontrolled pre-post evaluation of 274 adolescents (mean age 16.07) indicated that the prevention program increased knowledge, improved help-seeking attitudes and help-seeking intentions among the students, and did not produce iatrogenic effects. However, there were no significant changes with regard to help-seeking actions [18]. A study by Baetens et al. [17] examined differences between the programs Happyles and HappylesPLUS targeting NSSI in 651 Belgian school pupils (mean age = 12.85 years) using a randomized pre-post design. Happyles is a stepped-care prevention program which focuses on enhancing general mental well-being and social connectedness. It is based on an eclectic approach, which is grounded in positive psychology, cognitive-behavioral therapy, and problem-solving [19]. While Happyles consists of a general in-classroom education program, HappylesPLUS additionally incorporates an in-classroom one-hour psychoeducation module on NSSI. As with the aforementioned SOSI program, no iatrogenic effects were found. However, no significant difference emerged between the two groups regarding the incidences of engagement in NSSI, although pupils did report a reduced likelihood of possible engagement in future NSSI, and an increased emotional awareness was observed [17].

Consequently, there is not yet any universal prevention program which has been found to reduce NSSI. SOSI is similar to gatekeeper training for suicide prevention, and HappylesPLUS tries to reduce NSSI through 1 h of psychoeducation on the basis of a program that was not originally developed to address NSSI, even though studies in the field of universal prevention of suicidality show the best evidence for skills-based approaches [13, 20]. For example, the SEYLE study (Saving and Empowering Young Lives in Europe) compared the three most frequently employed types of suicide prevention (early detection/screening by professionals, gatekeeper training, skills-based group training) in a four-arm randomized controlled trial over a period of 12 months with 11,110 adolescents (mean age 15 years). According to the mentioned systematic reviews, only the interactive skills approach of a school-based universal prevention program YAM (Youth Aware of Mental Health) was associated with a significant reduction in severe suicidal ideation and incident suicide attempts compared with the control group [21]. As NSSI has been found to be a predictor of suicidality, the implication may be derived that the key to effective prevention lies in skills-based group training.

### Reason for the trial

NSSI has become not only a clinical but also a huge public health problem. Above all, it represents a high-risk marker for the development and persistence of mental health problems including high rates of morbidity and mortality, and causes substantial costs for the health system. While there are targeted prevention programs which have been shown to be effective, to date, only two school-based universal prevention programs exist (SOSI and HappylesPLUS), and these were found not to reduce NSSI. Thus, there is a gap between the necessity for universal prevention of this epidemic problem and the availability of such programs. As it has been proven that skills-based approaches in targeted prevention might change the tragic development of suicidality [14], increased efforts should be undertaken to develop universal prevention programs based on a skills-based approach in order to reduce NSSI in a school setting.

### Objectives and aims

The main objective of the present study is to evaluate a newly developed universal prevention program (“DUDE – Du und deine Emotionen / You and your emotions”) based on a skills-based approach in schools, in 3200 young adolescents (age 11–14 years). The effectiveness of DUDE will be tested in a cluster-randomized controlled trial (RCT), meaning that the participating schools (*N* = 16) will be randomly assigned to the respective conditions (see the “Procedure and randomization” section). All groups will receive a minimal intervention with a booklet called “Stress-free through the school day” as a mental health literacy prevention against burnout in school. The treatment group (*N* = 1600; 8 schools) will be divided into: treatment group 1 (DUDE will be conducted by trained clinical psychologists; *N* = 800; 4 schools) and treatment group 2 (DUDE will be conducted by trained teachers; *N* = 800; 4 schools). The active control group (*N* = 1,600; 8 schools) will only receive the mental health literacy prevention. The main outcome is the occurrence of NSSI within the last 6 months assessed by a short version of the Deliberate Self-Harm Inventory (DSHI-9) at the 1-year follow-up (primary endpoint; T3). We hypothesize that DUDE will show a lower incidence of NSSI compared to the active control group, demonstrating its superiority. In addition, secondary outcomes such as emotion regulation and suicidality, health-related quality of life, self-esteem as well as comorbid psychopathology, and willingness to change will demonstrate the superiority of DUDE over the active control group.

### Primary hypothesis

Participants in the treatment group (1 or 2) receiving DUDE will show a lower occurrence of NSSI within the past 6 months at T3 compared to students in the active control group.

## Methods/design

### Setting and recruitment

The present trial is a study from the newly founded German Centre of Prevention Research in Mental Health at the University of Wuerzburg in Germany. The aim of this center is to develop and implement existing prevention programs, evaluate their efficacy and effectiveness, and ensure their sustainability and broad dissemination.

In the present trial, we aim to include *N* = 3200 young adolescents (age 11–14 years) in Germany within a school-based setting with 16 schools. There are several reasons why the school setting is ideally suited for this trial: (1) First of all, NSSI is a phenomenon with epidemic proportions. Based on the high prevalence and the possible outcomes of NSSI, a school-based setting and population is reasonable in terms of universal prevention. (2) As mentioned, there is a need to further develop and evaluate universal prevention in the field of NSSI (see the “Background” section). (3) Targeted trials entail a selection bias, meaning that access is often limited to the location where the targeted prevention is implemented (mostly universities or clinics). Furthermore, adolescents will not be stigmatized during the allocation process. Hence, even if adolescents are suffering and undergoing emotional struggles but are afraid or reluctant to ask for help, they will eventually benefit from the participation in DUDE. Therefore, their risk of developing mental health problems in general, and engagement in NSSI in particular, might be decreased.

**Fig. 1:**
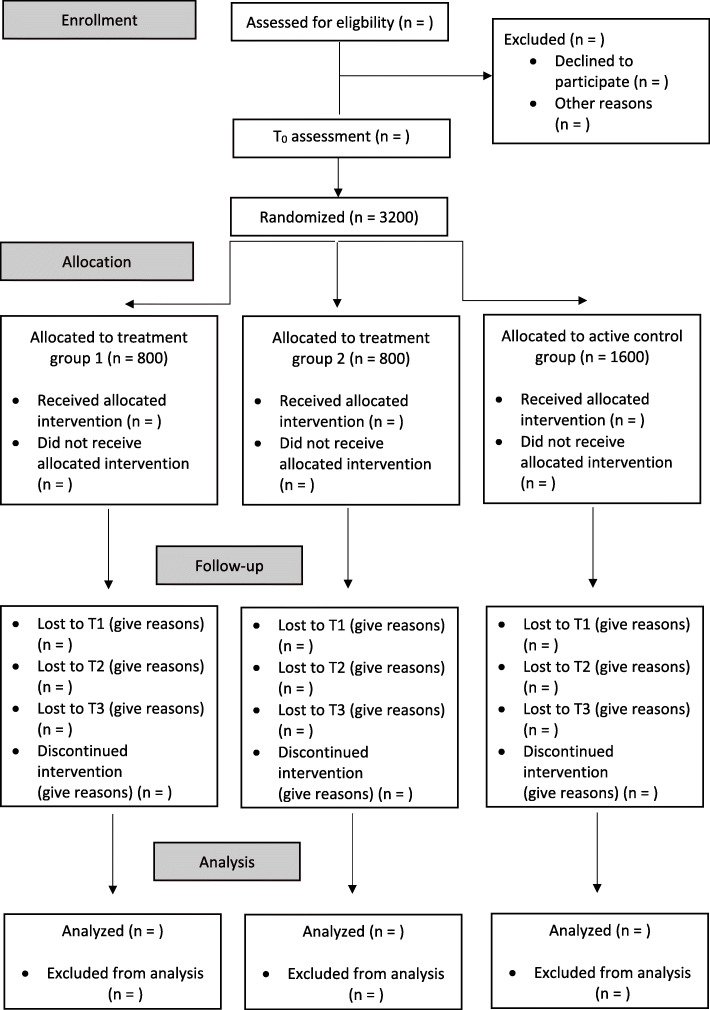
Study flow diagram in accordance to the Consolidated Standards of Reporting Trials (CONSORT)

**Fig. 2:**
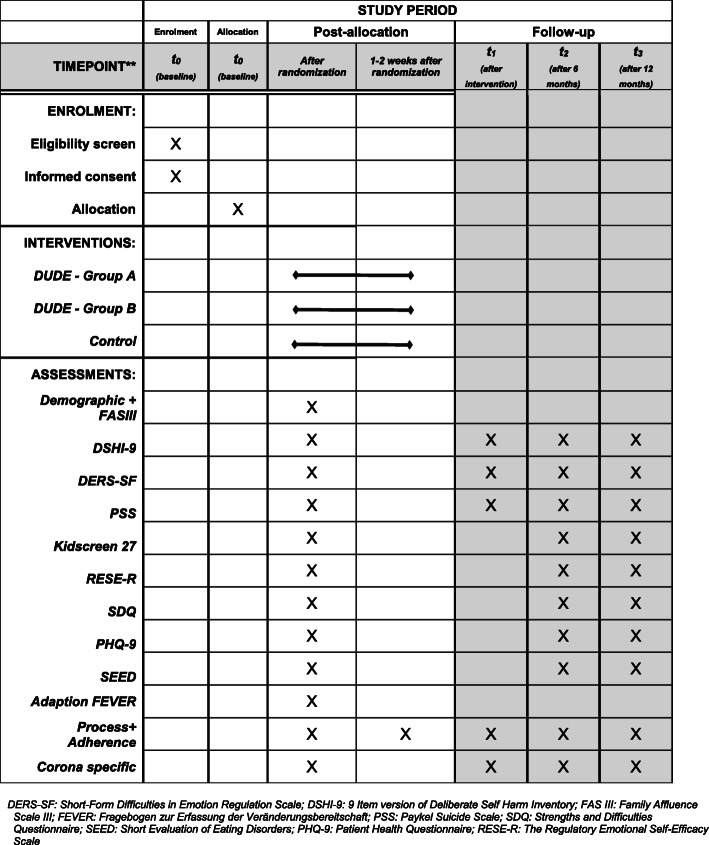
SPIRIT figure: Schedule of enrolment, interventions, and assessments

The recruitment of the 16 schools will occur in collaboration with the Bavarian Ministry of Education and Cultural Affairs. First, the schools will be selected at random by a staff member of the Ministry. Following this, the schools will be randomly assigned (cluster randomization) to the treatment group (1 or 2) or to the active control group using cluster randomization by an assigned external researcher who is not part of the study group. All eligible pupils of the participating schools will be offered the chance to participate in the study. The assessment time points of the study are before the start of treatment (baseline, T0), at the end of the treatment (T1), 6 months after the end of treatment (T2), and 12 months after the end of treatment (T3) in order to evaluate the efficacy of the universal prevention. The flow chart of the trial is shown in Fig. 1 and the trial schedule is depicted in Fig. 2.

### Inclusion and exclusion criteria

Eligible adolescents from the assigned schools will be asked to take part in the study. There are only two criteria for inclusion in the study: Participants must be aged between 11 and 14 years and in the 6th or 7th grade. We chose this age group because epidemiological data show that the end of childhood and the beginning of adolescence represents the period with the highest risk of onset of NSSI [22]. Accordingly, the first rise in incidence rates can be determined around the age of 14 years. Based on the rationale of universal prevention, a program should start before this period, keeping in mind that a universal prevention approach should start before a disorder develops [23]. Likewise, only two exclusion criteria will be applied: lack of informed consent from the adolescents or their relatives, and currently receiving individual psychotherapy because of a mental disorder. If participants report being in individual psychotherapy, to avoid stigmatization, they will still attend the training together with their classmates, and their data will be collected but saved separately. Thus, these participants will still be able to see information about their results, but these results will not be included in the data analysis. This procedure was chosen due to potential confounding effects between individual psychotherapy and the prevention program.

### Procedure and randomization

Prior to the start of the study, a feasibility stage has been completed, in order to check the following aspects: (1) iatrogenic effects: no harmful effects occurred; (2) feasibility check of DUDE and assessment measures: the adolescents enjoyed participating in DUDE; some of the instruments were adjusted as some pupils had difficulties understanding the questions; (3) Integrity of the emergency procedure: was not employed, but a simulation of an emergency took place. Furthermore, the communication and exchange with the two participating schools were excellent, and all parties were satisfied with the procedure. During this stage, two clinical psychologists provided DUDE, as the main aim was to check whether DUDE is accepted by the young adolescents. After this, DUDE was partially adapted with respect to some procedures or the length of certain exercises.

After the feasibility stage, the main study will start. First of all, the schools for the main study will be chosen by the Ministry of Education and Cultural Affairs. The respective school principals will then be contacted to ensure that the implementation of the study will run smoothly. This will be followed by a personal meeting with the teaching staff and the study team to inform about the rationale, aims, and study procedure. At this point, schools will not yet have been randomly assigned to a trial group. This should prevent any bias due to motivational effects during the recruitment. Once schools have given consent, pupils and parents will receive information about the study in the following order: (1) An information evening (1 ½ h) about the study will be held for parents and pupils. (2) Subsequently, parents and participants will once again receive all information in writing and the consent form via mail or e-mail. (3) To give the participants another opportunity to ask questions, information about the study will be provided again (during a regular lesson) before data collection, and the presence of the consent forms will be checked. The written and oral information will include the following: background and objectives of the study, information about the prevention program (DUDE) and minimal intervention, benefits and possible risks of participation, data collection procedures, and data security procedures. After parents and adolescents have received the required information, informed consent will be obtained. The parents and participants will confirm with their signature that they have read and understood the provided information and that they are willing to participate. Nevertheless, they will be able to withdraw from the study at any time without any consequences for subsequent school matters or medical care (see also the “Ethical issues and dissemination” section). Next, schools will be randomized. Before the baseline measurement, pupils will be checked for eligibility and the booklets (“Stress-free through the school day”) will be handed out. Seven days (± 3 days) after the baseline measurement, DUDE will conducted in treatment groups 1 and 2 and will be integrated into the normal school schedule.

Given the very large number of over 3000 participants, the measurements and execution of DUDE will be split into two time points. We aim to begin in November 2021, and the second round of assessment should take place around March 2022. However, due to the SARS-Covid-19 pandemic, delays are possible. To ensure that enough individual pupils give their consent to participate, the DUDE training will be integrated into the regular school day, during lesson time, to make participation more attractive to the students.

As mentioned above, to prevent bias, 16 schools will be randomly (cluster randomization, see the “Discussion” section) assigned to DUDE or to the active control group, with eight schools per group, using cluster randomization. Within the treatment group, there will be a secondary randomization into treatment groups 1 or 2. Enrolment, generation of allocation sequence, and assignment of schools will be carried out using the software SAS by an independent member of the institute not involved in the project. The schools will be informed about their group affiliation via e-mail and telephone call immediately after randomization. Nevertheless, blinding is not completely possible, as schools cannot be blinded to the different nature of the interventions. Blinding of the researchers is also not possible because the treatment group will receive DUDE in addition to the minimal intervention.

### Data assessment

Data will be collected and assessed in cooperation with the Institute of Clinical Epidemiology and Biometry, University of Wuerzburg, Germany. The baseline assessment (T0) will take place after randomization and 7 days (± 3 days) before the subsequent start of the treatments. At the end of DUDE or 5 weeks after receiving the minimal intervention (± 7 days), the second measurement (T1) will be conducted. Further assessments will ensue 6 months (± 7 days; T2) and 12 months (± 7 days; T3) after the end of DUDE. After every measurement, the data will be screened for potential suicidality (see the “Emergency procedure” section). At T2, only a selection of the assessment measures will be used (see Fig. 2). We chose this procedure in order to check for iatrogenic effects which could occur immediately after the prevention [24], whereas protective effects, or rather efficacy, in universal prevention is expected later [25]. Additionally, we will evaluate how appealing DUDE is to the adolescents, as this might have an impact on the effectiveness of the program.

### Assessment measures

T0 includes sociodemographic questions gathering information about gender, nationality, status of mental health treatment, current living situation, media consumption, and the socioeconomic status of the families, as measured with the brief version of the Family Affluence Scale (FAS III). The FAS III is a revised version of the Family Affluence Scale [26–28] which was validated in 7120 pupils from several European countries and appears to be a valid instrument to measure socioeconomic status [29]. In accordance with Good Clinical Practice (GCP), primary and secondary outcomes will be assessed.

#### Primary outcome

For the assessment of NSSI, we will use a modified version of the 9-item *Deliberate Self-Harm Inventory* (DSHI-9), which was used in the SEYLE study [12]. The original version of the DSHI, by Gratz, contains 17 items [30], and the DSHI-9 is an adaptation for adolescents by Lundh [31]. The modified version comprises the same facets on frequency, severity, and duration of NSSI as the 9-item version; however, self-injurious acts are combined in order to simplify and shorten the measure and to assess direct self-injury to one’s body surface only [32]. The internal consistency for the DSHI-9 lies between alpha = .66 and .85 [33].

#### Secondary outcomes

Emotion regulation will be assessed using the DERS-SF [34], an 18-item short version of the original 36-item *Difficulties in Emotion Regulation Scale* [35, 36]. The DERS-SF measures emotion regulation on six dimensions (Lack of Emotional Awareness, Nonacceptance of Emotional Responses, Difficulties Engaging in Goal-Directed Behavior, Limited Access to Emotion Regulation Strategies, Impulse Control Difficulties, Lack of Emotion Clarity). Cronbach’s alphas are .79–.91 for the subscales and 0.98 for the total score [37]. Thus, the DERS-SF is capable of measuring emotion regulation deficits to the same extent as the original DERS [34, 37].

The *Paykel Suicide Scale* (PSS) [38] is a short questionnaire consisting of five items exploring suicidal ideation and previous suicide attempts. A modified version with the following three response options will be used: “within the last two weeks,” “at some point in the past,” or “never”. Psychometric properties are difficult to find in the literature; however, the PSS has been used for many years to assess suicidality in adults and adolescents [39–43].

The *KIDSCREEN-27* will be used to assess participants’ general health-related quality of life (HRQoL). The scale was developed across Europe for use in children and adolescents (aged 8–18 years) [44, 45] and consists of 27 items measuring HRQoL on 5 scales (Physical Well-being, Psychological Well-being, Parents & Autonomy, Social Support & Peers, School Environment). It is a commonly used valid instrument with high internal consistency (Cronbach’s α range from .78 to .84) [44, 45].

The revised *Regulatory Emotional Self-Efficacy Scale* was developed to assess self-efficacy beliefs in the domain of emotion regulation (RESE-R) [46, 47]. It contains 12 items rated on a Likert scale from 1 (= not at all well) to 5 (=very well) and is composed of two subdimensions: One represents the perceived self-efficacy in expressing positive emotions (POS) and the other represents the perceived self-efficacy in expressing negative emotions (NEG). The negative dimension constitutes a second-order factor with two factors: self-efficacy in managing despondency/ distress (DES) and self-efficacy in managing anger/ irritation (ANG). Good internal consistencies for the individual areas have been reported (*α* = .68 to .79) [46].

To assess emotional and behavioral difficulties, or rather general psychopathology, the *Strengths and Difficulties Questionnaire* (SDQ) [48] with 25 items will be used. The SDQ collects information about problems and resources in the areas of emotional problems, hyperactivity/ attentional problems, problems with peers, behavioral problems, and prosocial behavior. A study evaluating the reliability and validity of the German version of the SDQ indicated that the questionnaire is valid for most clinical and research purposes [49]. Moreover, the SDQ shows good internal consistency (*α* = .81) [50].

For the assessment of depressive symptoms, the 9-item *Patient Health Questionnaire* (PHQ-9) will be used. The PHQ-9 measures depressive symptoms according to the DSM-IV criteria in a self-administered assessment, which has been found to have high sensitivity (89.5%) and good specificity (78.8%) [51]. The scale meets the clinical diagnostic criteria according to the ICD-10 (International Classification of Diseases, 10^th^ Revision) and has shown high sensitivity (87.1%) and good specificity (79.7%) among adolescents, with a good rtt = .87 and good internal consistency (Cronbach’s *α* = .83) [52].

The Short Evaluation of Eating Disorders questionnaire (SEED) [53] consists of six items and allows the calculation of severity indices of anorexia nervosa (underweight, fear of weight gain, distortion of body perception) and bulimia nervosa (binge eating, compensatory behavior, excessive overconcern with weight, and body shape). The questionnaire has good construct and criterion validity. The sensitivity to change also ensures that treatment changes can be assessed. The questionnaire has been used successfully in prevention studies in this age group [54]. We will measure eating disorder psychopathology because starving, vomiting, and binge eating are sometimes used as dysfunctional behavior in adolescents with problems in emotion regulation [55] and are strongly associated with NSSI [56].

#### Process and adherence variables

We assess process variables among pupils to determine whether and how much students liked DUDE. If pupils indicate that they liked the program, we assume increased motivation during participation, which could be associated with better transfer to everyday life. In addition, we survey the adherence of the trainers. It is important to check whether the manual is followed and how the success of DUDE is evaluated after each implementation. We assume that the adherence and the attitude of the trainers towards the program could also have an impact on the efficacy of the program.

In line with this rationale, additional items will be presented to evaluate both the DUDE program itself and the booklet (“Stress-free through the school day”). On a 6-point Likert scale, participants will rate six items regarding how helpful they found the different chapters of the booklet to be, and 10 items regarding how helpful they found the sessions of the training to be, whether they liked the implementation of the program and what they learned during the training.

Program adherence of the clinical psychologist or teachers will also be measured using a self-constructed instrument. Five items ask about how satisfied the teachers are with the sessions, whether they think the program was helpful, and about the cooperation of the students. Furthermore, to verify whether the coaches stuck to the manual, a checklist will be used to determine whether they implemented the specific contents of every lesson.

#### Confounding variables

The confounding variables control for whether students are open to the changes DUDE might bring about in their thoughts, feelings, and/or behavior (willingness to change) and how exposed they were/are to the SARS-Covid-19 pandemic-related consequences, as both factors could influence primary and secondary outcomes.

Willingness to change is increasingly cited as an important variable in health behaviors, and a predictor of treatment success: The *University of Rhode Island Change Assessment* (URICA) scale is used to measure patients’ motivation to learn and change [57]. Willingness to change is assessed according to four subscales (Precontemplation, Contemplation, Action and Maintenance) and the psychometric properties have been found to be good, with coefficient alphas from .79 to .89 [58]. In this study, a briefer and modified version of the German translation, called *Fragebogen zur Erfassung der Veränderungsbereitschaft* (FEVER [59];), will be used. This comprises 24 items which have been semantically adapted to match the prevention program and will assess the pupils’ willingness to change by asking about whether they are keen to participate, willing to try the strategies they are taught in the program, and their impression of the trainers [59].

Finally, ten questions regarding the SARS-Covid-19 pandemic will be asked, which explore stress factors, general mood, and well-being in families during the pandemic. These questions are from the Corona Health App, which was developed as part of a scientific cooperation between university partners, the Robert Koch Institute, and software companies (for detailed information see, https://www.corona-health.net).

### Interventions

*N* = 3200 participants in 16 schools (8 per group) will be cluster-randomized to the treatment groups, providing the minimal intervention as well as DUDE, and the active control group, providing only the minimal intervention. Table 1 provides an overview of the minimal intervention and Table 2 an overview of DUDE.

**Table 1:** Overview of booklet “Stress-free through the school day” goals, content, and targeting risk factors

Booklet unit	Goals	Content	Targeted risk factors
1. General information	• To increase knowledge and sensitivity regarding stress	• Definition of stress • Description of positive and negative consequences of stress • List of overload signs	• Improving the ability to identify stress and overloads (especially at school)
	• To deal with stress at school	• Different causes of school stress • Test: How stressed are you?	
2. How to prevent stress	• To gain techniques for efficient learning	• Tips to get started • Tips to avoid distraction • Tips for a better time management • Presentation of specific cognitive learning strategies	• Improving stress management • Strengthen body and mind • Internalizing suitable learning strategies
	• To strengthen resources and vitality	• Nutrition tips • Tips to establish healthy sleep habits • Tips to relax • Meaning of positive thoughts	
3. Getting help	• To seek help if necessary	• Overview and importance of the social network • Overview of unhealthy ways to deal with stress	• Lower inhibition threshold to seek help
	• To have contact addresses within reach	• Summary of possible points of contact

**Table 2:** Overview of DUDE goals, content, and targeting risk factors

DUDE unit	Goals	Content	Targeted risk/protective factors
1 – Feelings are like Waves	• To introduce to the topic / to commit to the program	• Introduction Video • Introduction and information regarding DUDE	
	• To build solidarity in the class	• Activating “Get to know Game” • Joint development of class rules	• Encouraging social support within the class
	• To improve one’s emotional perception and awareness	• Interactive group game regarding emotions • Exercise for the mindful perception of emotions	• Improving the ability to identify, differentiate and express one’s own feelings
2 – Overwhelmed by Feelings	• To improve awareness about stress and emotional tension	• Activating group game to show that everyone experiences stress • Guided group discussion to introduce the concept of “aversive emotional tension	• Improving stress management
	• To gain adaptive skills to deal with daily struggles	• Collection of stress tolerance skills in small groups • Class discussion of pros and cons of specific skills • Group game in which students train skills to prevent impulsive reactions	• Training impulse control
3 – Riding the Wave of Feelings	• To recognize the importance of emotion regulation	• Video about emotionally stressful events being handled maladaptive • Joint acquisition of the different stages of emotion regulation by referring back to the video	• Increasing the access and flexibility to different emotion regulation strategies
	• To improve one’s ability to regulate – especially negative - emotions	• Interactive exercises and role plays to train various mood-stabilizing skills	• Reducing negative affect
4 – What gives you boost?	• To learn to rely on oneself	• Group brainstorming about resources • Mindfulness-based exercise to train radical acceptance • Introspective exercise to become aware of own strengths and abilities	• Improving self-esteem and self-efficacy
	• To improve one’s interpersonal skills	• Group game in which the students must collaborate • Group exercise to train benevolent and respectful interaction with one another	• Counteracting bullying by an improved class climate and enhanced interpersonal abilities
5 – Jump into the wave’	• To anchor knowledge in everyday life	• Repetition of program contents and acquired strategies • Definition of intentions: “What do I want to implement in everyday life?”	• Improving self-efficacy by encouraging self-responsibility

#### Minimal intervention

Within the minimal intervention, information material (a booklet called “Stress-free through the school day”) on the subject of how to handle stress, especially at school, will be distributed. This form of universal prevention follows the principles of a mental health literacy prevention program. The booklet provides general information on stress, its possible negative outcomes, and ways to prevent it. Moreover, cognitive techniques are outlined which help to make learning more efficient and consequently less stressful. For detailed information on the program goals and content, see Table 1.

#### DUDE

The development of the program was preceded by a systematic review of the literature. Targeted prevention, which significantly reduces NSSI and improves mental health, is based on elements of cognitive behavioral therapy (CBT) and dialectical behavior therapy (DBT) [14, 60]. The main goal of these approaches is to find alternative behaviors for NSSI against the background of an improved emotion regulation. Deficits in emotion regulation, especially in terms of coping with uncomfortable feelings and relief from emotional stress or pressure, are the most common reasons for NSSI [61]. A recently published meta-analysis also revealed a significant association between emotion dysregulation and NSSI, with emotion dysregulation encompassing limited access to regulation strategies, non-acceptance of emotional responses, impulse control difficulties, and difficulties engaging in goal-directed behavior [62].

These factors should be addressed in an effective universal prevention and a skills-based approach seems reasonable to achieve this (see the “Background” section for further information).

DUDE includes five 90-min units conducted at weekly intervals. The didactic methods consist of skills coaching, including interactive methods (experience-based approaches, group discussion and role plays) and theme-specific cartoons (videos) to anchor the content. Homework has to be completed after every lesson in order to integrate the content into the adolescents’ daily routine. A tandem of a male and a female clinical psychologist or teacher (depending on treatment group) will conduct DUDE. In accordance with the metaphor of Kabat-Zinn [63] “You can’t stop the waves but you can learn to surf,” meaning that emotions cannot be prevented but one can learn how to deal with them, DUDE is set up as an imaginary surf camp. The five units are (1) “Feelings are like waves,” (2) “Overwhelmed by feelings,” (3) “Ride the wave of feelings,” (4) “What gives you boost?,” and (5) “Jump into the wave.” For detailed information on the program goals and content, see Table 2.

### Emergency procedure

If the participants report serious suicidal thoughts in one of the measurements (assessed with the PSS, especially item 4 “Have you ever reached the point where you seriously considered taking your life or perhaps made plans how you would go about doing it?,” answering “Yes, within the last two weeks”), there will be an immediate (within 24 h) report back to their family. The family will be informed about the situation by one of the principal investigators and an appointment with a personal contact will be made at the Department of Child and Adolescent Psychiatry, University Hospital of Wuerzburg, or in one of the admission clinics with which the study team is cooperating. During this appointment, suicidality will be examined and, if necessary, a treatment offer will be made. If adolescents indicate serious suicidal thoughts within a unit of DUDE, psychologists or teachers will telephone the psychiatrist on duty to clarify the situation in a first step, and subsequently inform one of the principal investigators, as needed. Over the entire duration of the study, participants and their caregivers will be able to contact the Department of Child and Adolescent Psychiatry, University Hospital of Wuerzburg, in the case of mental health problems. This will also be indicated in the information letter.

### Coaches

In treatment group 1, DUDE will be administered by psychologists. The psychologists have completed an undergraduate psychology degree, and at least one member of each tandem providing DUDE in schools is currently in training to become a clinical psychologist. In treatment group 2, DUDE will be implemented by trained teachers. All coaches will have taken part in a 1-day course to prepare them for conducting the program and will additionally receive an implementation manual in order to standardize the procedure. Furthermore, there will be the possibility for weekly supervision if requested from the coaches.

### Sample size

The primary endpoint is the incidence of NSSI measured by the dichotomized DSHI-9 at T3. We assume an incidence rate (newly detected cases in the last 12 months) of 2.31% in the control group and 0% in the treatment group. This assumption is based on data from a longitudinal study by Baetens et al. [64], which reported a prevalence of 3% in the control group at age 12–13 years and a prevalence of 5.31% later at age 14–15 years. We hope that due to our prevention program, no new cases will occur in the treatment group and assume a stable prevalence rate of 3%. In the active control group, an increase from 2.31 to 5.31% in 1 year can be assumed based on the data of Baetens [64].

To account for the need for cluster randomization in our study, we need to calculate an intraclass correlation coefficient (ICC). We assume an ICC of 0.015. As there was no ICC available for NSSI, this assumption is based on data from two studies of binge drinking behavior in adolescents [65, 66]. Comparability between NSSI and binge drinking can be expected, as both represent maladaptive strategies for coping with one’s own feelings or for dealing with personal stress.

We plan to recruit 160 pupils per school (four classes per grade, 6th and 7th grade (age 11–14 years)). A two-sided unpooled *z*-test for cluster-randomized studies will be used to test the null hypothesis of equal proportions. A sample size of 160 pupils per school and eight schools per group yields a power of 80% to detect a difference between the proportions of 2.31%. The significance level will be set at 5%. Including a dropout rate of 20%, 200 pupils per school and a total number of 3200 pupils need to be recruited. The sample size was estimated using PASS 15.05.

### Statistical analysis

The primary hypothesis will be addressed by using a univariate generalized linear mixed-effects model with logit link function for the probability of newly detected NSSI (measured dichotomously) at T3. To model the correlation within schools, schools will be included as random effects.

As secondary analyses, the generalized linear mixed model for the primary outcome will be adjusted for potential sociodemographic confounders (sex, socioeconomic status, mental stress due to the COVID-19 pandemic). In addition, we will adjust the baseline measure NSSI, although our analysis will only consider the incidence rate (newly detected NSSI in the last 12 months). Furthermore, based on the distribution of the secondary outcomes, differences in secondary outcomes between the two groups (treatment and intervention) will be compared. We will analyze emotion regulation (DERS-SF), suicidality (PSS), quality of life (KIDSCREEN-27), self-efficacy (RESE-R), general mental health (SDQ), depression (PHQ-9), and eating disorder pathology (SEED) using univariate mixed-effects models, and with non-parametric tests if necessary due to the data material.

Missing data and individuals who withdraw from the trial will be handled using an intention-to-treat (ITT) approach. All participants randomized will be considered in the analysis. In the case of dropout or missing data, we will use the multiple imputation method.

### Data safety

Participant confidentiality will be ensured by generating unique study identifiers unrelated to participants’ real names. The collected data and the study identifiers will be kept at the data-holding facility and can only be decoded by the principal investigator through a list in which the names and study identifiers are provided together. This list will be stored in a locked cupboard. Decoding can only take place if suicidality occurs in a respective participant (see the “Emergency procedure” section) or the participant would like to be informed about the results of the assessments.

The Institute of Clinical Epidemiology and Biometry, University of Wuerzburg, will operate independently from the study site to control and supervise the data collection and safety. Data will be collected using the EDC system REDCap. This is a secure web application for building and managing online surveys and databases, which is specifically geared to support online and offline data capture for research studies. Data storage and transfer will only be possible on the secured servers of the University of Wuerzburg, and access will be password-protected and strictly limited to authorized personnel only. For data analysis, the anonymized data will be securely transferred to servers of the Chair of Clinical Epidemiology and Biometry, hosted by the University Hospital of Wuerzburg. Computerized assessments guarantee the highest level of data integrity and quality, i.e., missing data will be minimized. However, as with paper-and-pencil questionnaires, human error can occur during data entry. Access to the data via REDCap allows the continuous monitoring of data collection including immediate response in the case of emergency, i.e., suicidality, as well as restoration of all previous states. A Distributed Replicated Block Device (DRBD) will provide synchronous replication of all data during data entry on two separate servers. In addition, full and incremental backups will be conducted following a predefined back-up plan.

### Harms

In view of the non-invasive universal prevention, the risk for the participants is considered marginal. Comparable studies (i.e., SEYLE study or SOSI) showed no iatrogenic effects such as an increase in suicidality or NSSI. Irrespective of the randomization, participating pupils will benefit from a universal prevention that protects against the development of psychopathology and especially NSSI and suicidality. However, an increase in NSSI and suicidality cannot be completely ruled out. Hence, an emergency procedure has been installed in order to help conspicuous pupils (see the “Emergency procedure” section). Nevertheless, there is no obvious risk for participants. The independent Institute of Clinical Epidemiology and Biometry, University of Wuerzburg, will provide expert advice regarding all aspects of data quality. This means that the data could be checked immediately after data entry for adolescents who are potentially at risk, ensuring that the Department of Child and Adolescent Psychiatry, University Hospital of Wuerzburg, can provide them with the necessary help as quickly as possible. Beyond this, the ethics committee and the Ministry of Education and Cultural Affairs will be informed about adverse events, such as suicidal acts, and the further course of the study will then be coordinated with these entities.

The principal investigators will inform surrounding psychiatric hospitals about the study in advance. Information about where to find help as well as necessary contacts will be publicly available on the study homepage and on the information letters given to the pupils beforehand, ensuring that all participants know how to access emergency contacts. Additionally, we will provide consulting addresses and the study staff will also be available by telephone and mail. If any adolescent feels overwhelmed during the data collection or the training, another emergency protocol is in place to offer help right away. In addition, we will be in close contact with the respective educational psychologists throughout the duration of the study to ensure that psychiatric/psychotherapeutic help can be easily provided if needed.

### Ethical issues and dissemination

The study will be conducted in accordance with the Declaration of Helsinki and the rules for physicians of the Bavarian State Medical Association (“Bayrische Landesärztekammer”) in their currently valid version. Participation in the study is voluntary, and all adolescents and their guardians will need to sign an informed consent form. Consent can be withdrawn at any time without stating a reason and without any individual disadvantage for subsequent school matters or medical care. In the case of study withdrawal, previously collected data will be deleted if desired unless data have already been included in the analyses or identification of the individual participant data is no longer possible. The participant list from which pupils can be identified will only be available to the school principal and will be destroyed after T3. In this way, we can assure that data can be fully anonymized. The study protocol has been approved by the ethics committee in Wuerzburg (127/19-me) and the Ministry of Education and Cultural Affairs (IV.7-BO5106/200/12).

In the case of relevant protocol modifications, the ethics committee and the Ministry of Education and Cultural Affairs will be informed immediately and an amendment will be submitted. Access to the protocol and information for the public is ensured through the registration and regular update of the trial in the German Clinical Trials Register (DRKS) (DUDE - the prevention program for schools to reduce self-injurious behavior by enhancing stress tolerance and emotion regulation; DRKS00018945; registration date 01 April 2020).

All confidential information will be subject to the rules of medical confidentiality and in line with the requirements of the European, Federal and State Data Protection Act (Europäische Datenschutzverordnung (EU-DSGVO), Bundesdatenschutzgesetz (BDSG), Landesdatenschutzgesetz (LDSG)). The data will be stored and processed in a pseudonymized manner. No third parties will gain insights into the original data. As reported above, participants can only be identified in the case of emergency or they would like to be informed about the results of the assessments.

The trial results will be disseminated through journal publications and scientific meetings, as well as a broader dissemination if there is evidence of the efficacy of DUDE. The study protocol will be made available to all participating schools. As it concerns data from underage pupils, we are not allowed to make the data set accessible to third parties according to the ethics committee and Ministry of Education and Cultural Affairs. The analysis code is provided as supplemental material each time the results are published. This broad dissemination will occur in cooperation with the KKH and further evaluations of effectiveness beyond a randomized control trial and comparable to stage III or stage IV studies in GCP. The use of professional writers is not intended.

## Discussion

NSSI is above all a high-risk marker for the development and persistence of mental health problems, encompassing high rates of morbidity and mortality and causing substantial costs for the health system. It is a common and highly recurrent behavior that peaks in adolescence [11, 12], and has been reported to be the fifth most frequent health risk in adolescence [1]. There is an urgent need to counter these developments and potentially tragic long-term consequences for young adolescents. What we do know is that targeted approaches significantly reduce NSSI and improve mental health, but access to such approaches is limited due to a lack of resources and specially trained physicians. A universal prevention program may help to overcome this matter and ensure an exhaustive protection to counteract this problem of epidemic proportions. However, so far, only one uncontrolled study (SOSI) has examined the effects of a universal prevention program for young adolescents [18] and on study examined the efficacy via a randomized controlled trial with a pre-post design [17]. Thus, this is the first RCT to develop and evaluate a universal prevention program with follow-up measurements to prevent NSSI. The present study, and especially DUDE, entails several advantages compared to previous work. First, we have consciously chosen not to employ any psychoeducational elements, as these have triggered iatrogenic effects in other universal prevention programs [24, 67]. Second, we provide a program addressing emotion regulation in accordance with effective psychotherapy (e.g., DBT) or targeted prevention programs (e.g., “cutting down”) in the field of reducing NSSI and suicidality over the emotional pathway. A third advantage is the implementation of DUDE in schools. On the one hand, adolescents spend most of their time at school and have to use emotion regulation skills on a regular basis. This facilitates the transfer to the students’ real life. On the other hand, as the school setting is capable of providing training for everybody, a large number of pupils can benefit, and most importantly, no stigmatization of the affected adolescents will occur. Fourth, bullying, as an important risk factor for mental health problems [68], is also addressed in DUDE by interactive exercises. This promotes cohesion within the group and strengthens solidarity.

### Limitations

Despite the mentioned advantages, potential limitations of the study design should be acknowledged. First, in accordance with the Ministry of Education and Cultural Affairs, to maximize practicability, we have chosen a cluster (school-wise) randomization procedure, which is common and recommended in the study of school-based prevention programs [25, 69, 70]. Nevertheless, this randomization procedure differs from other studies, and especially psychotherapeutic studies, which randomize participants by subject or class. We will seek to protect the study from contamination through the randomization procedure. Randomizing at the school level ensures that participants, and particularly those from the control conditions, are unable to obtain material or information covered in the prevention condition due to contact with each other [69]. Second, we are trying to standardize the implementation of DUDE in the treatment groups through training and the manual such that high manual fidelity can be ensured. However, we are aware that in treatment group 2, teachers are unlikely to adhere to our manual 100% due to their many daily responsibilities. This will potentially have an impact on the efficacy of our program. We will attempt to monitor standardized implementation with measures of adherence (see the “Process and adherence variables” section) after each lesson, as in psychotherapy studies. However, it is not certain that this will work. It is important to determine if there are group differences between the different trainers (clinical psychologists vs. teachers) in the implementation of DUDE, as this could affect the efficacy of the program. Ultimately, the implementation of DUDE by teachers is hugely important for subsequent dissemination. Third, we must ask whether DUDE is superior to targeted prevention, because universal prevention usually achieves low effect sizes. Perhaps it would make more sense to identify pupils with high-risk or emerging self-injurious behavior using a brief questionnaire and refer them to targeted interventions that have already been shown to be effective. However, the problem with this procedure is that disclosure of psychopathological behavior in the school setting may still lead to stigmatization of affected students. Fourth, our findings must take into account the SARS-Covid-19 pandemic and its impact on pupils’ mental health. If the pandemic-related conditions that led to psychological and behavioral problems among adolescents are reversed and a “normal” daily routine is possible again, this could also lead to an improvement in mental health and be falsely associated with the efficacy of DUDE. However, the randomized controlled trial design should prevent such misconceptions, and the additional measurement of SARS-Covid-19 burdens (see the “Confounding variables” section) should control for the influence of this factor on efficacy. Nevertheless, due to the current pandemic-related high mental stress and decreased quality of life among children and adolescents, we believe it is imperative to implement universal prevention programs to improve emotion regulation and reduce maladaptive behaviors in order to help those affected and actively contribute to improving the current situation for young people.

DUDE is tailored to diminish the incidence of NSSI and prevent adolescents from the possible long-term consequences thereof (e.g., suicidality). It provides easy access for adolescents due to its implementation in a school environment and is cost-effective. Furthermore, DUDE is a comprehensive approach which tries to improve mental health through the pathway of improved emotion regulation.

### Trial status

Protocol version 2, 27 June 2020. The trial is scheduled to be completed on May 2023 with T3. Recruitment will begin in October 2021 and recruitment will be completed in April 2022. https://www.drks.de/drks_web/navigate.do?navigationId=trial.HTML&TRIAL_ID=DRKS00018945

## Data Availability

Not applicable.

## Abbreviations

BDSG: Bundesdatenschutzgesetz
CBT: Cognitive Behavioral Therapy
DBT: Dialectical Behavior Therapy
DERS-SF: Difficulties in Emotion Regulation Scale- Short Form
DSHI-9: Deliberate Self-Harm Inventory - 9 items
DRKS: Deutsches Register für Klinische Studien
DUDE: Du Und Deine Emotionen
EU-DSGVO: Europäische Datenschutzgrundverordnung
FAS III: Family Affluence Scale III
FEVER: Fragebogen zur Erfassung der Veränderungsbereitschaft
ICC: Intraclass correlation coefficient
ITT: Intention-to-treat
LDSG: Landesdatenschutzgesetz
NSSI: Non-suicidal self-injury
PSS: Paykel Suicide Scale
PHQ-9: Patient Health Questionnaire- 9-item version
RCT: cluster-randomized controlled trial
REDCap: Research Electronic Data Capture
RESE-R: Regulatory Self-Efficacy Scale
RKI: Robert Koch Institute
SARS: Severe Acute Respiratory Syndrome
SDQ: Strengths and Difficulties Questionnaire
SEED: Short Evaluation of Eating Disorders
SEYLE: Saving and Empowering Young Lives in Europe
SOSI: Signs of self-injury
STAR: Self-injury: Treatment, Assessment, Recovery
URICA: University of Rhode Island Change Assessment
WHO: World Health Organization
